# ‘I Didn’t Even Recognise Myself’: Survivors’ Experiences of Altered Appearance and Body Image Distress during and after Treatment for Head and Neck Cancer

**DOI:** 10.3390/cancers13153893

**Published:** 2021-08-02

**Authors:** Chandrika Gibson, Moira O’Connor, Rohen White, Melanie Jackson, Siddhartha Baxi, Georgia K. B. Halkett

**Affiliations:** 1 Curtin School of Nursing, Faculty of Health Sciences, Curtin University, Perth, WA 6845, Australia; g.halkett@curtin.edu.au; 2 WA Cancer Prevention Research Unit (WACPRU), School of Population Health, Faculty of Health Sciences, Curtin University, Perth, WA 6845, Australia; M.OConnor@curtin.edu.au; 3 Department of Radiation Oncology, Sir Charles Gairdner Hospital, Nedlands, WA 6845, Australia; Rohen.White2@health.wa.gov.au; 4 GenesisCare Perth Radiation Oncologist, Wembley, WA 6014, Australia; melanie.jackson@genesiscare.com; 5 GenesisCare Gold Coast Radiation Oncologist, John Flynn Hospital, Tugun, QLD 4224, Australia; siddhartha.baxi@genesiscare.com

**Keywords:** head and neck cancer, body image, disfigurement, altered appearance, psychological adaptation, cancer survivorship, communication skills, trauma, compassion

## Abstract

**Simple Summary:**

In interview data collected from 21 people diagnosed with head and neck cancer in the previous six years, participants reported adequate procedural preparation but little or no preparation related to appearance. Body image distress contributed to psychosocial issues for many people, negatively impacting their adaptation to altered appearance. The main themes included; Preparation (sub-themes: Decision-making; and Preparation for Altered Appearance); Altered Appearance (sub-themes: Weight Loss; Face, Skin and Hair Changes; and Reconstructive Surgery); and Consequences (sub-themes Reactions from Others; Adapting to Altered Appearance). Current practice provides information pre-treatment about many aspects of coping; however, the subject of appearance is not routinely addressed. Communication skills training for health professionals that improves their comfort and sensitivity in discussing and conveying compassion around issues of altered appearance, body image, and trauma, is needed to decrease suffering for survivors, support healthy adaptation to living with altered appearance, and increase patient satisfaction with health care.

**Abstract:**

Purpose: Preparation for head and neck cancer treatment is focused on practicalities of treatment. Little or no time is spent prior to treatment discussing aesthetic results of treatment or the psychosocial impact of living with an altered appearance after treatment. The objective of this study was to explore the experiences of survivors of head and neck cancers, with a focus on the psychosocial impact of altered appearance. Methods: A qualitative research approach based on social constructionist theory was used. Twenty-one semi-structured interviews were conducted with survivors of head and neck cancer who had been diagnosed in the previous six years. Thematic analysis was used to identify themes. Results: People diagnosed with HNC reported feeling rushed into treatment, with adequate procedural preparation but little or no preparation related to appearance. The main themes included: Preparation (sub-themes: Decision-making; and Preparation for Altered Appearance); Altered Appearance (sub-themes: Weight Loss; Face, Skin and Hair Changes; and Reconstructive Surgery); and Consequences (sub-themes Reactions from Others; Adapting to Altered Appearance). Conclusions: Body image distress related to altered appearance, contributed to psychosocial issues for many people diagnosed with head and neck cancer. Current practice provides information pre-treatment about many aspects of coping; however, the subject of appearance is not routinely addressed. Communication skills training for health professionals that improves their comfort and sensitivity in discussing and conveying compassion around issues of altered appearance, body image, and trauma, is needed to decrease suffering for survivors, support healthy adaptation to living with altered appearance, and increase their satisfaction with health care.

## 1. Introduction

Head and neck cancer (HNC) is a particularly distressing cancer [1,2], with approximately 35% of survivors experiencing adjustment issues, anxiety or depression [3,4]. Individuals diagnosed with HNC not only face a potentially life-threatening diagnosis but must endure treatments that often result in altered appearance. Research on survivors of HNC has shown that altered appearance is a source of psychological distress [1,5]. Changes in appearance and function can be the result of surgery [6] and chemo-radiotherapy [7]. Surgical treatment to the face and neck has been found to create negative changes in auto-perceived body image in 1/3 of survivors with head and neck cancer (*n* = 36) [8]. In a survey of people with HNC undergoing surgery, 75% reported feeling concerned or embarrassed by bodily changes following treatment [9]. Facial alterations from head and neck disease, and facial transplants have been described as traumatic and, alongside complications affecting functions such as eating and speaking, contribute to feelings of embarrassment and shame, leading to isolation and negative health consequences [6,10,11,12,13].

The highly visible head and neck region is closely associated with sense of self, as faces are a primary social communication tool. Temporary and lasting changes to appearance affect self-concept [12] and survivors need to reconstruct their self-concept and body image after treatment [14]. Gili suggests that in altered facial appearance, the face ceases to be the absent background to perception and gives rise to disrupted perceptions, sensations and observations [14]. This sense of altered self -concept makes it harder for survivors to return to their pre-cancer identities, roles and activities [15] resulting in social isolation [16]. Altered appearance can result in body image disturbance in adults who have been treated for HNC where attributes include: self-perception of a change in appearance and displeasure with this perceived change; a decline in an area of function; and psychological distress regarding changes in appearance and/or function [17]. Adjusting to life with an altered appearance is affected by factors including the extent of the altered appearance, the emotional value placed on appearance, cultural and social norms, the survivors’ comfort in managing interactions, and support from their closest community [16,18,19,20]. Reconciling changes in appearance may not resolve spontaneously [4,21] and typically requires supports and interventions.

Psychosocial interventions have proven effective for conditions of altered facial appearance [22] including in HNC [7]. However, almost all of these interventions have been offered reactively post-treatment [23,24,25,26]. In a retrospective cohort study of HNC survivors (*n* = 1992) it was found that a pre-treatment psychoeducational intervention resulted in better oncologic outcomes [27], however, appearance related concerns were not addressed. Rifkin states that ‘*While there is likely a complex interplay between physical, cultural, and psychosocial factors and successful adaptation to facial disfigurement, a deeper understanding of these factors might help guide development of interventions that facilitate adaptation to facial disfigurement*.’ [28]. It should be noted that much of the literature uses the term ‘disfigurement’; however, because some survivors of HNC report the term to be stigmatising [14], in this paper we will refer to altered appearance instead.

The aim of this study was to explore the experience of survivors of head and neck cancer, with a focus on the psychosocial impact of altered appearance. This project is part of a larger study conducted to understand the psychosocial education and support needs of people with HNC and the experiences of health professionals who care for them [29].

## 2. Materials and Methods

### 2.1. Design

An in-depth qualitative design was adopted using semi-structured interviews. Social constructionist theory underpinned the design and provided a basis for understanding how realities and views of the world are experienced, interpreted and constructed through an individuals’ relationships and via a combination of complex interactions with society, and the meanings that individuals attribute to such interactions [30].

### 2.2. Participants

To be eligible for this study, participants needed to be over 18 years of age, English-speaking, diagnosed with head and neck cancer in the last six years, regardless of HPV status, and treated in Australia. Twenty-one participants were recruited from cancer centres in tertiary hospital settings, private practice, and not-for-profit cancer support organisations. Snowball sampling was used to recruit further participants. See Table 1 for demographics.

### 2.3. Procedure

Recruitment of a convenience sample began with the first author explaining the study to HNC health professionals at hospital multidisciplinary team meetings. Health professional (HP) interviews were also conducted [29]. The HPs recruited relevant participants by providing information sheets and requesting their permission to provide contact details to the researcher, who contacted them by email or phone based on their stated preference. Two of the contacted potential participants declined to participate due to poor health. 19 interviews were conducted face-to-face, and two via an online video conferencing platform. No interviews were repeated. Most interviews took place in participants’ homes with one at a treatment centre and two online. A partner was present during one interview, otherwise participants were alone with the interviewer. Interviews were 45–75 min (mean = 61 min, SD = 9.40). All interviews were digitally audio-recorded and transcribed verbatim. Transcripts were not returned to participants.

Interviews were conducted by the lead author using a semi-structured interview guide which had been reviewed by HPs and pilot tested by a consumer. Topics included diagnosis, pre-treatment information, treatment experiences, prognosis, unmet needs, emotional wellbeing and the impact of HNC on roles and relationships. Participants were asked questions and prompted to elaborate and provide examples from their experience to ensure data were grounded in their realities. The interview guide is available as Supplementary Material.

### 2.4. Data Analysis

Data was entered into NVivo12 (https://www.qsrinternational.com/nvivo-qualitative-data-analysis-software/home (accessed on 18 June 2021), an application for qualitative data management. Following the six steps described by Braun and Clarke [31], inductive thematic analysis was conducted by the first, second and last authors. This entailed: (1) familiarisation with the data by repeated reading of transcripts, (2) generation of initial codes line-by-line, (3) identification of recurrent themes, (4) theme checking throughout single transcripts, followed by the entire dataset, (5) creation of labels and definitions for emergent themes, and (6) utilisation of codes and themes to construct findings.

### 2.5. Rigour

Data collection and analysis were conducted concurrently until data saturation was reached and no new information emerged [32]. A reflexive diary, commonly used in social constructionism, was used to provide a consistent and systematic documented account of the process and context, including interviewer’s reflections on body image issues. The first author, a PhD candidate and experienced patient educator, undertook sensitive interview training prior to conducting the interviews. The co-authors participated in interpretation of data, with discussion amongst the research team until consensus on themes was reached, contributing to the dependability of the findings [33]. Reporting was informed by the Consolidated Criterion for Reporting Qualitative Research (COREQ) checklist [34]. The COREQ checklist is available as Supplementary Material.

## 3. Results

**Figure 1:**
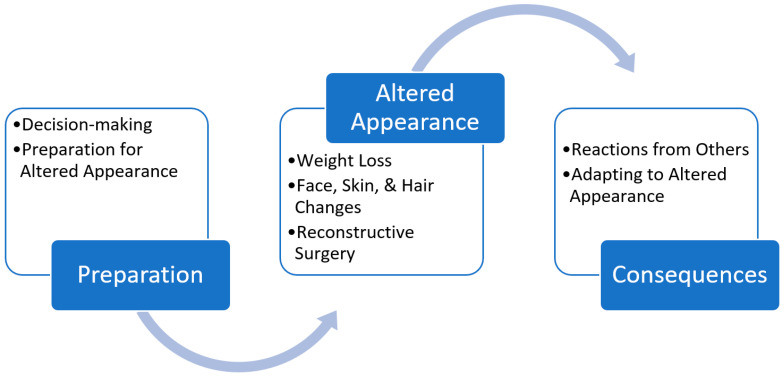
Themes and Sub-themes.

Survivors offered a range of insights about their experiences of being diagnosed with HNC, the impact treatments had on their appearance and function, and the social interactions and communications that influenced their social construction of meaning. Their constructions were articulated via these key themes and sub-themes: Preparation (sub-themes: Decision-making; and Preparation for Altered Appearance); Altered Appearance (sub-themes: Weight Loss; Face, Skin and Hair Changes; and Reconstructive Surgery), and Consequences (sub-themes: Reactions from Others; and Adapting to Altered Appearance) (Figure 1).

### 3.1. Preparation

Survivors expressed high levels of satisfaction with pre-treatment preparation in terms of what procedures were expected, and all but one participant felt adequately prepared for treatment. However, they felt unprepared for and traumatised by changes to their appearance, indicating there was inadequate preparation and a lack of informed, shared decision-making. Most survivors described placing trust in their treatment teams, *‘I just trust what you are going to do. And that’s all there is to it’* PID21. Sub-themes relating to preparation included: Decision-making; and Preparing for Altered Appearance.

#### 3.1.1. Decision-making

Treatment options were presented to patients, however the appearance related consequences were not explicitly discussed. One participant reported that he felt so traumatised following appearance-altering treatment, he regretted his treatment decision. Describing a scenario of cancer recurrence, shaking his head at the idea of undergoing treatment a second time, he said ‘*I would have to think really hard now if I really wanted to do that again.*’ PID16

#### 3.1.2. Preparation for Altered Appearance

Some survivors expressed fear at learning that body parts would be removed to reconstruct the features and organs of their face. Hearing the description of how the surgery would be completed was a shock to most people, for one participant it led to a panic attack:

*‘I heard they were cutting out every part of the upper part of my mouth and they would replace that with parts of my leg, and it was beyond my understanding that they could do that inside my mouth. I felt so confronted and I was starting to shake, and I was getting pins and needles, so my husband just took me for a little walk up the corridor and back, and I had some water. I was dealing with the visceral shock of my mouth being invaded.’ *

PID13

PID7 initially believed he had a sinus problem requiring cosmetic surgery, but then found it was nasal cancer, requiring complete removal of his nose. This participant expressed that treatment moved quickly and discussed starting radiation therapy unprepared:

*‘There was no preparation, they just said about the treatment and of course I wasn’t expecting it to be as uncomfortable as it was the first time.’*

Sometimes treatment didn’t go to plan and was more extensive than patients’ expected. When asked if HPs had prepared him for the appearance related results of surgery one participant said:

*‘They told me I would lose my teeth, but he did say they were going to save the eye. This eye was not able to be saved though as complications arose.’ *

PID17

Some survivors asked more questions, but when it came to appearance, most felt it was not worthy of raising and tried to gather information from sources other than the treatment team. For some participants seeking information on the internet was distressing:

*‘I Googled some things that they just said that I was going to have done and you’d only have to look at one picture and you just freak and turn it off.’ *

PID16

In the absence of more direct communication, survivors were left to deduce what was happening, for example:

*‘I had to meet the prosthodontist for him to take extremely careful views of what my smile looked like at the time-so in other words, get the look of what (participant’s name) is before we do this to her.’ *

PID13

Despite having concerns, the participant still didn’t know what to expect in terms of appearance after surgery:

*‘I had no idea whatever about what I was going to look like… and I felt vulnerable in anticipation of that.’ *

PID13

### 3.2. Altered Appearance

Participants felt underprepared for appearance changes that occurred during and after treatment. For some survivors looking in the mirror was a shock:

*‘I didn’t even recognise myself. I didn’t even know who I was. I looked in the mirror and I didn’t even see myself. That’s how different I was.’* PID20, while others avoided or delayed looking at the results of surgery, *‘it took me about two weeks to actually have a look at where my nose was.’*

PID7

It was common to express that aesthetic outcomes were less important than clinical results and yet, some participants were distressed and disappointed with the loss of their pre-HNC appearance:

*‘I often said to them, “Look, I understand there’s people that you could be operating on who’ve got critical cancer and this is really all cosmetic,” but I was offered the world and then suddenly it all filters back down to zero.’ *

PID17

Sub-themes describing aspects of altered appearance included: Weight Loss; Face, Skin and Hair Changes; and Reconstructive Surgery.

#### 3.2.1. Weight Loss

Common side effects related to radiotherapy for HNC are xerostomia (dry mouth), thickened saliva, altered taste and mucositis (painful mucous membranes). These side effects combine to make eating challenging [35]. As a result, many participants described losing weight rapidly:

*‘I’ve got dry mouth, I’ve got thick saliva, I‘ve got occasional pain in my throat similar to after the tonsillectomy-type pain, feels like scabs are there… I lost maybe 10 kg in a week.’ *

PID11

Many survivors underwent concurrent chemotherapy which caused nausea and/or vomiting, further contributing to low appetite and under consumption of calories:

*‘They kept giving me injections for nausea… but I couldn’t eat; I didn’t want to eat’. *

PID16

Dietitians were frequently involved to provide intervention to manage the side effects of treatment and prescribing high calorie, nutrient dense food and supplements. For some participants it was the first time a health professional had encouraged them to try to put on weight. Some survivors observed that they had been heavy all their lives, frequently cautioned by health professionals against weight gain, so they had mixed feelings about losing weight:

*‘I think (it) is ironic because all my life I’ve been told to lose weight–but there’s this huge focus on keeping your weight up, because if you don’t there are all sorts of things that can go wrong like your mask won’t fit properly and the treatment won’t work as well.’ *

PID10

Survivors who started with higher body mass, typically lost weight rapidly:

*‘I’ve gone from 120 kilos to 79 kilos. I look like a skin and bone man…my face was so withdrawn, I was like a skeleton…I have to sit down in the shower now and I’m looking at my legs going, “Jesus Christ, there’s nothing there.”.’*

PID20

While those who were smaller to begin with, became emaciated:

*‘I’m only a small build so I looked like something out of a concentration camp.’ *

PID16

For some the significant weight loss came during the weeks of radiotherapy and chemotherapy:

*‘When I went to treatment I was 60 kilos and when I finished my treatment, I was 38 kilos.’*

PID8

For others, major weight loss occurred after completing radiotherapy:

*‘I might have lost two or three kilos during treatment, but the big weight loss was when the mucositis comes in and your membranes are all so sore, you can’t swallow, you can’t eat, you can’t sleep. I lost 20 kgs.’ *

PID3

#### 3.2.2. Face, Skin, and Hair Changes

Appearance changes related to treatment toxicity were commonly experienced, with participants reporting erythema, lymphoedema, hair loss, and osteoradionecrosis. All but one participant received radiotherapy to treat their HNC, and experienced erythema-inflamed, red and irritated skin. Survivors described the outward appearance of red skin as well as the sensations of discomfort it caused:

*‘It was like somebody taking a blowtorch and just burnt…all my neck and my face.’*

PID17

PID14 suffered dry, irritated, itchy skin so uncomfortable he could not tolerate his shirt touching his neck, leading him to self-isolate:

*‘Pretty uncomfortable really. I couldn’t go visiting. I went to my daughter’s on Tuesday. I was there about half an hour, and I had to come home. I just couldn’t talk; I was tired. And I wanted to take my shirt off.’*

Chemo-radiotherapy caused hair loss including facial hair, and many participants observed temporary loss of eyelashes and eyebrows:

*‘I’ve lost my eyebrows, but they’re starting to come back. My eyelashes, I’ve lost them, but my facial hair now comes back a lot darker.’*

PID7

Face shape also changed for some participants such as PID18 who experienced unsightly swelling:

*‘I looked like I had 10 chins and he (doctor) said, “That’s just where everything’s been attacked and nuked. It’ll gradually go down.”‘ *

PID18

Radiation treatment has a cumulative effect and side effects often worsen at the end of treatment and after treatment completion. PID4 observed symptoms worsening:

*‘Particularly the last couple of weeks, was quite horrible because it was that cooked and a lot of temperature and a lot of blistering on the neck.’*

While most participants experienced the worst skin changes immediately following treatment, some experienced late effects including osteoradionecrosis:

*‘I got this pretty much straight after the radiation; it started deteriorating my jaw. (It took) five years all up before the jaw actually broke...the jawbone just got so infected and broke and my face was out like a balloon.’ *

PID16

#### 3.2.3. Reconstructive Surgery

Surgical removal of HNC commonly requires follow up reconstructive surgery for both appearance and function restoration. Participants described aspects of their complex surgeries that caused them distress, such as PID13 who was not informed that she might have leg hair growing inside her mouth:

*‘The fact that the skin that’s where my teeth are now, is from my leg, means I get hair there sometimes.’*

Sometimes these free flaps and grafts did not work-for PID17 the promise of a palate took multiple attempts:

*‘(they used) my stomach muscles. That was quite a good palate, it really hung tight actually and then they put a bit of scapula with a bit of bone in, in the muscle…they thought they’d be able to screw teeth into that possibly… that failed after a week so then they went in and put some muscle out of my thigh in there and luckily that took okay.’*

There were commonly delays for those survivors who required follow-up cosmetic surgery:

*‘That’s about a two hour operation, but unfortunately, because it’s not life-threatening, I have to go on the waiting list.’ *

PID7

PID16 required multiple surgeries to recreate one side of his face:

*‘They took the jaw from there down to here somewhere and then they took the tibia bone out of my leg and put it in there and then they put a chain, like a bicycle chain-type thing around there for it to heal, but they also had to cut this side of my neck as well to bring all the blood vessels and nerve endings and everything over to this side because radiation had actually killed everything on this side of the face.‘*

Altered appearance included the area used to rebuild, where people were left with large scars and functional deficits:

*‘I find it really hard at the moment walking because where they took the bone out of the leg that seems to be more of a problem than the neck and they said that would happen, where they take skin and bone and things is normally the last to heal.’ *

PID16

### 3.3. Consequences

Survivors felt acutely aware of the reactions of others to their altered appearance. From interactions with hospital staff, to seeing themselves in the mirror for the first time, and interacting with partners, parents, children and communities, survivors found it challenging to adjust to their new appearance:

*‘People say they don’t notice it, but I do and I hate it more than anything’. *

PID8

Sub-themes used to describe the consequences of altered appearance included: Reactions from Others; and Living with Altered Appearance.

#### 3.3.1. Reactions from Others

Some survivors found the reactions from others to be one of the most distressing aspects of their HNC experience: *‘The lack of education with other people and their insults is something that I had to learn to deal with.* ’ PID6

*‘I haven’t got good self-esteem. To go out, I’m very conscious of people watching me, looking at me. Before you used to look [at] somebody and you’d make eye contact with somebody, that’s fine, but now it’s eye contact then their contact goes to your face, your neck, and I’m quite aware of it.’ *

PID16

One participant had become aware of the way people were reacting to her emaciated appearance and decided to say something:

*‘Bad enough people stare at me already because I look like some kind of anorexic walking around…I said, “I’ve just had radiation and chemo for cancer. I actually can’t eat anything.’ *

PID8

Other participants reflected on comments made from others, such as PID6, who also became emaciated, experienced stigmatising comments, and isolated herself so others wouldn’t see her:

*‘For a whole year in this house I never went out… It all started with a family dinner. When I walked in one of the men said, “Oh my God, (name), you’re so skinny. You really need to put on some weight.” And that started something for me….I was devastated.’ *

PID6

For others it was not the reactions from others that hurt, rather the feeling that being unrecognisable to friends was isolating:

*‘I’d have people walk up talking to like me and (partner), family friends walk up and start talking, they were talking to her, and they were sort of looking at me and looking away. They didn’t even know who I was. And then (partner) would go, “Aren’t you going to say hello to (participant’s name)?” and they were like, “Oh.” Nobody knew what to say to me.’ *

PID20

Family members sometimes did not recognise survivors after treatment:

*‘I didn’t see what I looked like, but nobody recognised me–my face was out here and it was green and (daughter) wouldn’t believe (partner) when she said, “That’s your dad there.” “No, that’s not Dad.” And my mother-in-law came. She took one look and went outside and burst out crying.’ *

PID17

PID16 thought that his altered appearance was so confronting, it put his marriage under pressure:

*‘You can understand these people that–you’ve got a married couple whatever and then she’ll just go or he’ll go because– it’s a nightmare for them… sometimes my wife would come to the hospital and I’d see her change colour; she’d just go white and have to go outside.’*

A sense of being stared at led to social withdrawal for some survivors. When PID7 first received a prosthetic nose, he tried venturing out:

*‘I went out a couple of times, but I felt uncomfortable because people were really staring at you because it was white, it stood out.’*

Later, his prosthetic nose was altered to look more natural:

*‘I said, “That’s really good, but it’s too new.” So I went back and said “Can you do some more colouring into it and put a bit other stuff in,” which he did.’*

This participant observed increased social isolation due to friends and colleagues avoiding seeing him in person:

*‘They’d pick up the phone and ring you, but they don’t actually come and see you.’*

#### 3.3.2. Adapting to Altered Appearance

Many survivors who experienced significant altered appearance described a feeling of loss–of the future they had imagined, and of a past version of themselves. For many it was important to their recovery to come to a realisation that ultimately appearance is not what matters, and the richness of their inner lives could not be altered by changing their appearance, for example PID7 who stated: *‘I’m still the same person as what I was before the operation.’* Through interactions with others, these constructions helped them adapt to their altered appearance.

PID17 rationalised that he is no longer young and further surgery aimed at cosmetic improvement would be futile:

*‘Intellectually and I think in my heart now I see that it would be ridiculous. I’ve already had three 12 h-plus operations on this side of my face and, it’s not like I’m 25.’*

Others have reassured him:

*‘A lot of people, friends, even women friends, have said, “You’re still the same person. It doesn’t matter what you look like.”’*

PID6 felt that people could see her strength as well as her fragility:

*‘I think people probably see me differently. They thought I was a tough cookie before, but they probably look at me and say that I’m quite a strong person… People look at me differently so I feel a bit different, although there is that negative thing of how they feel about me, my weight and how I look, but they also know that I have definitely done it tough and I’m okay still.’*

Returning to social and recreational activities was an important part of recovery for survivors, even when difficult conversations arose:

*‘When I go to my local tavern, people ask me what’s happened? I told the bar staff when I had the plastic on and they were, “Bloody hell, you’re brave coming out like that.” I said, “Well, what do you want me to do?” You can’t sit in the house.’ *

PID7

## 4. Discussion

This study provides an understanding of the psychosocial trauma of altered appearance, and the construction of the world experienced by people undergoing treatment for HNC. Analysis of the HNC survivors’ narratives demonstrates the perceived lack of HP led communication, preparation, screening, and support for people undergoing treatment which is likely to result in altered appearance. All participants reported appearance-related changes, and many experienced lasting changes, which challenged their sense of self due to the social importance of the visible face and neck area. It was through interactions with hospital staff, health professionals, family and strangers that survivors constructed meaning from their experiences. While survivors found ways to adapt, many were re-traumatised by distressing social interactions. These adjustment difficulties are similar to Glassey et al.’s research which found younger women undergoing bilateral prophylactic mastectomies experienced psychological distress related to altered appearance post-surgery. Interestingly, those who had psychological support pre- breast cancer surgery in the study by Glassey et al. adjusted better and reported a more positive body image [36]. This approach of pre-surgery psychological support would be useful for people undergoing surgery for HNC. Every interaction is potentially supportive, so it is important that all health professionals build on their communication skills by undertaking focused training which addresses communication around psychosocial issues of altered appearance and prepares patients to adapt following treatment.

Changes in weight and body composition brought up issues with body image for some survivors. Many participants experienced negative feelings about weight loss which aligns with previous literature, where weight loss is reported to be associated with depression, distress and stigma [37,38]. A novel finding is that some participants were pleased to have lost weight, despite their health professional’s advice to maintain body mass. The high degree of focus on dietary intake caused distress for some participants, and there could be a risk of triggering disordered eating. This could be mediated with appropriate screening for body image concerns and eating disorders [38].

Survivors were reticent when requesting information about aesthetic reconstructive surgeries or prostheses as they believed their appearance related concerns may be perceived as superficial. This finding supports earlier work by Fingeret et al. [39] who found that cancer patients were reluctant to raise body image concerns with health professionals. Earlier research found that, rather than being driven by vanity, or even self-consciousness, concerns about altered appearance stemmed from a loss of sense of self, and fear of being stigmatised by others [40]. The interactions between survivors and health professionals typically focused on dysfunction such as pain and inability to eat and did not address altered appearance or body image issues. Furness et al. also found HPs underestimated or neglected psychosocial aspects of rehabilitation following facial surgery for reconstruction due to traumatic injuries or to remove cancer [41]. The clinical implications of lack of preparation for altered appearance include lower patient satisfaction with care, and greater demand for supportive care services post treatment. Some survivors regret having had treatment, potentially disengage from further screening and may decline future treatment if they had a cancer recurrence. This is similar to treatment regret due to altered appearance which has previously been found in women who undergo prophylactic mastectomies [36].

Most participants did not feel prepared for their altered appearance, and their first social interactions with family members sometimes caused further distress due to the reactions of hospital visitors. As they returned to their communities, interactions with friends, family, acquaintances and strangers were frequently experienced as intrusive, insensitive, distressing and added to problems of adaptation. This finding supports the work of Furr et al. [6] who found that problems with altered appearance following face transplantation are rooted in social interactions. Some participants anticipated rejection or disgust due to their changed appearance and would have understood if their partner wanted to leave the relationship. Many others voiced concerns for their loved ones related to their changed appearance. This finding supports work in the field of altered appearance related to orofacial gangrene which found that survivors expected partners to be repulsed [13]. This has implications for psychosexual wellbeing, intimacy, and caregiver support, and there is a need for further research in this area.

Survivors described self-isolation and avoidance of interactions, which reflects Newell’s fear-avoidance model of psychosocial difficulties following altered appearance [42]. The tendency to socially withdraw due to altered appearance was also found in a study of partners and survivors who both described changed social and recreational lifestyles, with some survivors withdrawing from social interactions because they did not want to be seen [43]. A number of authors have found that the responses of people close to the survivor have an impact on their adjustment, with negative reactions impeding emotional adaptation [44], and positive social interactions building confidence [15]. Withdrawal from social engagement leads to isolation, loneliness and a loss of confidence, as voiced by our participants and reflected in the literature [15,41].

The effects of radiation treatment on skin were both uncomfortable and unsightly, leading survivors to socially withdraw. Male participants reported feeling sensitive to both how it felt, and how it looked, which contrasts with Rennie et al. who found that men with HNC prioritise function, and distance themselves from concerns about appearance [21]. Our findings align more closely with a study of head and neck and lung cancer survivors, which found stigma was higher for men than women, and highest when altered appearance was greatest [11]. Stigma is generally likely for people with altered appearance regardless of gender [45]; however, it is possibly harder for men to disguise an altered appearance as they tend not to use makeup, scarves or other accessories which could hide the area. Being able to camouflage visible altered appearance has been found to lower distress for female survivors of HNC [46], however, Graboyes et al. point to the inability of cosmetic interventions to effectively address complex psychosocial aspects of body image disturbance [7].

Participants who had reconstructive surgery often required multiple surgeries and described looking very different to their internalised image of themselves, even unrecognisable. Looking in the mirror elicited negative emotional responses, with some survivors avoiding the mirror for weeks after treatment. This has been termed ‘mirror trauma’ [47] and has been explained as a significant autonomic nervous system shock reaction via the polyvagal response [48]. Earlier research has found similar shock at first sight [41,49], with those who avoid looking in the mirror, likely to experience delayed adaptation [50]. Across a variety of cancer types, a similar theme of concern around future appearance when undergoing reconstructive surgeries can be found. In cancer survivors seeking counselling, regardless of age, sex or cancer type, concern with future appearance was the strongest predictor of counselling enrolment [51]. Our findings point to the need for compassionate, non-judgmental interpersonal interactions to support acceptance of altered appearance. Further research on appearance focused interventions for HNC survivors and those who care for them, including HPs and family carers [52] is needed. Interventions should build on communication skills such as recognizing and responding to emotion, and incorporate health professional reflexivity, self-compassion, and understanding of the impact of cancer treatments which alter appearance.

### 4.1. Limitations

A strength in this study is that survivors of both HPV and non HPV related HNC were included. Seventy-one percent of participants were male, which is also a strength, as while HNC affects more men than women, health research where participants self-select, often means more female participants are included. The inclusion of an indigenous participant represents 5% of participants compared with 3.3% of the Australian population (ABS 2019). All other participants (95%) were Caucasian. Given the lack of cultural diversity of participants there may be cultural differences that were not explored.

### 4.2. Practice Implications

Health professionals who prepare people with HNC for treatment need to provide personalised education about altered appearance and could consider screening for distress and referring to pre-surgery psychological support. Where body image distress or appearance related concern is identified by patient disclosure, administering valid and reliable body image screening tools [53,54,55] prior to treatment could be used to trigger discussion and referral to Psychologists or other supportive care professionals. Regardless of explicit mention of body image or appearance concerns, referrals to psychosocial support should be made routinely prior to treatments expected to alter appearance. All HPs who work with people with HNC could benefit from increased awareness of the psychosocial impact of altered appearance, and training in order to offer focused, trauma-sensitive, compassionate communication prior to and after treatment. Future research could also include peer support groups and family carers, in order to develop interventions that address the social context of survivorship.

## 5. Conclusions

Body image disturbance and living with altered appearance lead to psychosocial distress for many people diagnosed with head and neck cancer. Current practice provides information pre-treatment about many aspects of treatment, however, the area of appearance is commonly not addressed. Providing greater information, screening, and preparation for altered appearance may influence treatment decisions, and is important for patient satisfaction and empowerment. Feeling unprepared for altered appearance as a result of treatment contributes to the experience of HNC as traumatic. Psychological assessment and support pre-treatment is recommended, especially when treatment is likely to result in altered appearance. Communication skills training for health professionals that improves their comfort and sensitivity in discussing and conveying compassion around issues of altered appearance, body image, and trauma, is needed to decrease suffering for survivors, support healthy adaptation to living with altered appearance, and increase their satisfaction with health care.

## Figures and Tables

**Table 1:** Participant Demographics.

**Age**	Min 34, Max 79	Mean 64 years	SD 10.75
**Gender**	Males 15	Females 6	Other 0
**Time since diagnosis**	Min 60 days, Max 1827 days	Mean 646 days or ~21.5 months	SD 713.00
**Diagnosis**	HPV (P16+)	Non-HPV	Unknown
	47% 10/21	33% 7/21	19% 4/21
**Treatment Received**	Surgery	Radiation therapy	Chemotherapy
	43% 13/21	95% 20/21	76% 16/21
**Race**	Caucasian	Aboriginal	Other
	95% 20/21	5% 1/21	0
**Education Level**	Less than school grad	School Grad	Beyond Year 12 qualification
	33% 7/21	24% 6/21	38% 8/21

## Data Availability

The data used to support the findings of this study are included within the article.

## Supplementary Materials

The following are available online at https://www.mdpi.com/article/10.3390/cancers13153893/s1, CORE-Q Checklist, Interview Guide.
