# Supplementary material for: ‘I Didn’t Even Recognise Myself’: Survivors’ Experiences of Altered Appearance and Body Image Distress during and after Treatment for Head and Neck Cancer
Source: Cancers (Basel). 2021 Aug 2;13(15):3893. doi: 10.3390/cancers13153893 (PMC8345413; doi:10.3390/cancers13153893)
Supplement: Supplementary file 1 [file cancers-13-03893-s001.zip › cancers-1286576-suppl/cancers-1286578-Interview Guide.pdf]

Interview Guide for People Diagnosed with Head and Neck Cancer (HNC)

Introduction

1. We are interested in understanding your experience of being diagnosed with HNC. Perhaps we could start with your account of your experiences with HNC so far?
2. Can you tell me about the treatments you have received so far?

Information Needs

3. Overall, what sort of information have you found most useful? Why was this?
4. Can you identify what information has helped you to reduce your fears and/or concerns during consultations? What sort of information was most useful initially?
5. So far, who has provided you with information?

Info re Diagnosis

6. What information have you been provided with about HNC?
7. How prepared do you feel for what is likely to happen in the future? What information has helped?

Info re Treatment

8. What information has been provided about treatment so far? Was this information helpful? Why/why not? How has it been useful?
9. At the moment, how well informed do you feel about your treatment?
10. Are you concerned about anything?

Questions relating to treatment (ask for each treatment received)

11. Did you feel adequately prepared for …. treatment?
12. What assisted you in preparing for treatment?
13. Who provided you with information?
14. Did you feel anxious or worried about receiving treatment?
15. What aspects concerned you?
16. Was it like you expected?

Info on Prognosis

17. Can you tell me about your prognosis?
18. What information about your prognosis has been provided? Was this information helpful? Why/why not?

19. How much of this information do/did you understand?
20. Was it discussed openly and in a sensitive way? Explain
21. Do you feel that you were told the right amount of information about prognosis? Too much?
22. Did you feel the timing of this information was appropriate?

In general

23. What type of information have you needed during this time?
24. Are there things happening to you that you feel you don't understand?
25. Have you accessed the information that doctors have provided to you? What information has been useful?
26. Have you independently accessed additional information? What about? Where? When? Why?
27. Which information sources have you found most useful/beneficial? Why? – written, video, verbal.
28. How do you feel about accessing information from the internet? Have you accessed the internet to gain information about radiation therapy?
29. Would you have preferred information to have been provided in a different way or using a different type of media? e.g. videos, education sessions etc.
30. When was information provided to you? Did you feel the timing was appropriate? Would you have preferred to receive information earlier/later?
31. Do you have any unmet information needs? What are they?
32. Have you had to ask for any information at a particular time? What sort?
33. What sort of information do you need now?

We are going to talk about your feelings and experiences now, so please remember that if any questions make you feel uncomfortable you can simply opt not to answer and we will move on to another topic.

Emotional and Practical support needs

34. How did you feel when you were first diagnosed?
35. Do you feel that you have assumed the role of patient? If yes, when did you take on the role?
36. Did being diagnosed with HNC change any of the other roles you play? e.g. Partner, employee, employer, recreation
37. Can you identify who has provided you with support since your diagnosis?
38. Do you feel like you are getting the support you need?
39. Have your support needs changed over time? Can you tell me more about when you did or didn't need various forms of support?

40. Do you feel like you can get additional support for yourself if/when you need it? Can you identify additional support needs you may have in the future?
41. Have you accessed support from other sources outside of the hospital? If so, what? Did you find these sources helpful? Why/why not?
42. Have you needed any practical or physical supports in the home? If so, what? Have you required financial assistance during this period? Has this need been adequately met?
43. Do you feel that the currently available patient support resources are adequate? If not,
    a) How could they be improved?
    b) What additional resources/supports do you think would be useful?

Self –Care and Psychological Wellbeing

44. Are there any practices that you have undertaken since learning of your condition that assist you in feeling good about yourself?
45. Has your physical appearance been altered by the disease and/or treatments? If so, what has that been like for you?
46. Have any of your relationships been impacted by your experience of HNC?
47. If you could rate your level of distress on a scale with one being very low and ten being very high, what number would you assign to your experience so far?
48. Have there been any positives to come out of your experience so far?

Thank you

Similar questions will be used at each of the different time points during the study.
